# The Role of B Cells in Primary Progressive Multiple Sclerosis

**DOI:** 10.3389/fneur.2021.680581

**Published:** 2021-06-07

**Authors:** Jameson P. Holloman, Robert C. Axtell, Nancy L. Monson, Gregory F. Wu

**Affiliations:** ^1^Department of Neurology, Washington University in St. Louis, St. Louis, MO, United States; ^2^Department of Arthritis and Clinical Immunology Research, Oklahoma Medical Research Foundation, Oklahoma City, OK, United States; ^3^Department of Microbiology and Immunology, Oklahoma University Health Science Center, Oklahoma City, OK, United States; ^4^Department of Neurology and Neurotherapeutics, University of Texas Southwestern, Dallas, TX, United States; ^5^Department of Immunology, University of Texas Southwestern, Dallas, TX, United States; ^6^Department of Pathology and Immunology, Washington University in St. Louis, St. Louis, MO, United States

**Keywords:** B cell, multiple sclerosis, immune pathogenesis, inflammation, primary progressive multiple sclerosis

## Abstract

The success of ocrelizumab in reducing confirmed disability accumulation in primary progressive multiple sclerosis (PPMS) via CD20-targeted depletion implicates B cells as causal agents in the pathogenesis of PPMS. This review explores the possible mechanisms by which B cells contribute to disease progression in PPMS, specifically exploring cytokine production, antigen presentation, and antibody synthesis. B cells may contribute to disease progression in PPMS through cytokine production, specifically GM-CSF and IL-6, which can drive naïve T-cell differentiation into pro-inflammatory Th1/Th17 cells. B cell production of the cytokine LT-α may induce follicular dendritic cell production of CXCL13 and lead indirectly to T and B cell infiltration into the CNS. In contrast, production of IL-10 by B cells likely induces an anti-inflammatory effect that may play a role in reducing neuroinflammation in PPMS. Therefore, reduced production of IL-10 may contribute to disease worsening. B cells are also capable of potent antigen presentation and may induce pro-inflammatory T-cell differentiation via cognate interactions. B cells may also contribute to disease activity via antibody synthesis, although it's unlikely the benefit of ocrelizumab in PPMS occurs via antibody decrement. Finally, various B cell subsets likely promulgate pro- or anti-inflammatory effects in MS.

## Introduction

Multiple Sclerosis (MS) is the most prevalent chronic demyelinating disorder of the central nervous system (CNS) affecting more than 2 million people worldwide and over 700,000 people in the United States (1). There are multiple different subtypes of MS. Most common is the relapsing remitting MS (RRMS) subtype that affects the vast majority of MS patients. Approximately 85–90% of patients present with RRMS (2), which is characterized by relapsing and then remitting neurological deficits without progressive disability between relapses. In later stages, RRMS patients may exhibit ongoing worsening without obvious remission, termed secondary progressive MS (SPMS). Roughly 36–60% of patients who first develop RRMS will go on to develop SPMS, on average 10 years after disease onset (3, 4). A less common subtype, primary progressive MS (PPMS), is characterized by gradual worsening of neurological function from disease onset without evidence of remission. Approximately 10–15% of patients with MS have PPMS (2). Of all the MS subtypes, PPMS has the worse prognosis, with patients reaching much higher levels of disability compared to patients with RRMS and SPMS (5). The pathophysiologic mechanisms leading to these distinct clinical phenotypes in MS subtypes is an area of ongoing research. The pathological hallmarks of MS are inflammation, demyelination, remyelination, and neurodegeneration occurring either focally or diffusely in the brain and spinal cord (6). These features are present in all MS subtypes, although in PPMS and SPMS there is a predominance of diffuse low level inflammation, slowly expanding pre-existing lesions, and a more intact blood brain barrier when compared to RRMS (7).

**Figure 1:**
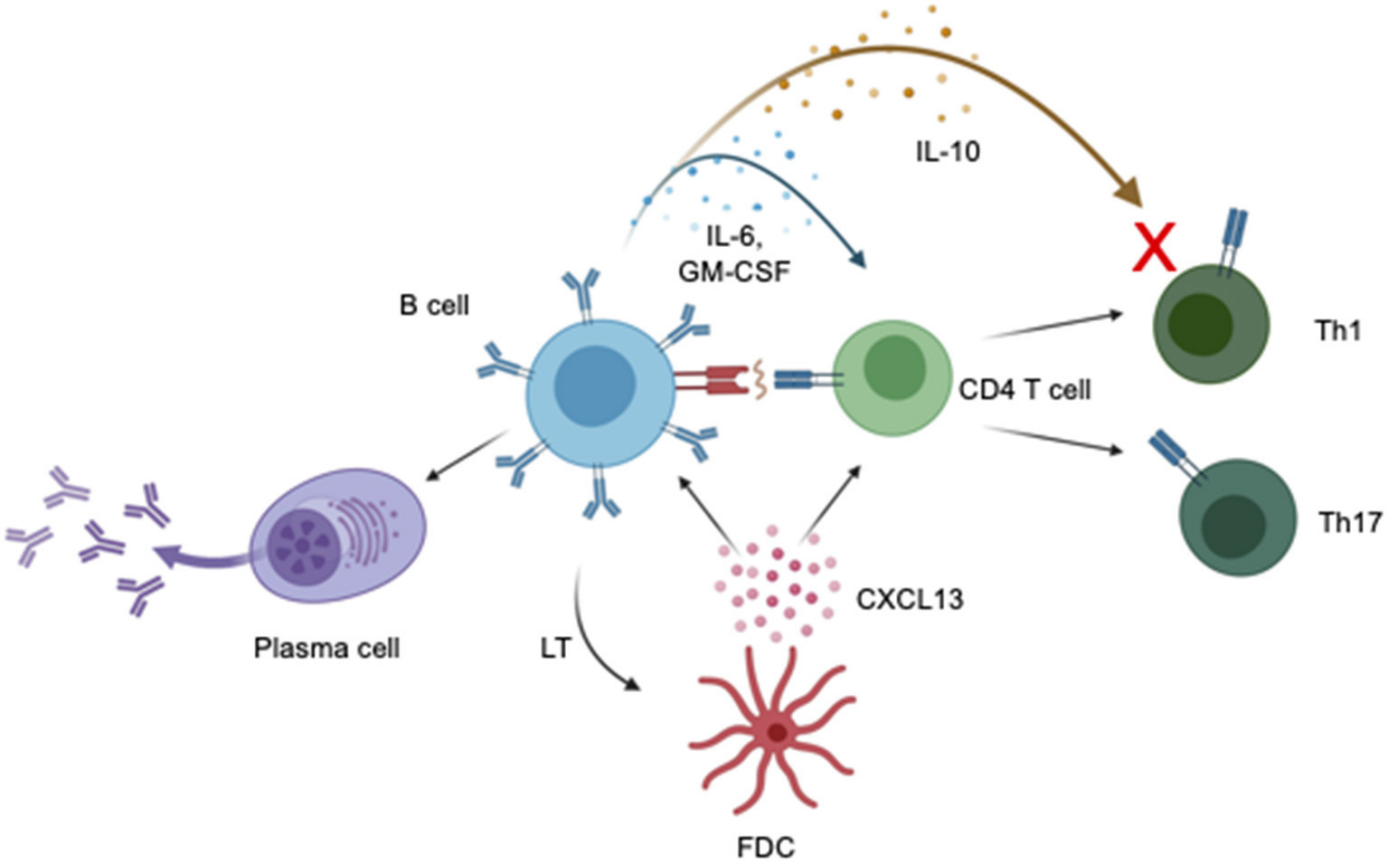
Impact of B cells on PPMS pathogenesis. Production of cytokines influences the function of CD4 T cells, including promoting and suppressing inflammation. Production of the cytokines IL-6 and GM-CSF can induce differentiation of CD4 T cells into Th1 and Th17 T cells which can then cause CNS damage. The cytokine IL-10 is believed to decrease activity of Th1 effector T cells and reduce neuroinflammation in EAE and MS. Decreased IL-10 production by B cells may result in increased neuroinflammation in MS. B cells induce T cell activation and differentiation into pro-inflammatory T cell subsets via antigen presentation via the tri-molecular complex of MHCII, antigen, and T cell receptor. B cells are capable of differentiating into antibody secreting cells which produce antibodies capable of directly damaging the CNS. Binding of lymphotoxin (LT) by follicular dendritic cells induces secretion of CXCL13 which may serve as a chemoattractant for B cells and T cells, increasing lymphocyte infiltration into the CNS. Created with BioRender.com.

B cells have been implicated in the pathology of MS through the presence and diagnostic significance of oligoclonal bands (8–11), an increased concentration of unique B cells subsets in the periphery and CNS of MS patients (12–15), and the formation of CNS ectopic lymphoid follicles (16–18). B cells may contribute to disease progression in PPMS through cytokine production, antigen presentation and antibody synthesis. A summary of the mechanism of action of B cells in the immunopathogenesis of PPMS is shown in Figure 1. Further, the effect of B cells in MS is likely subset-dependent with some B cells exerting an anti-inflammatory effect (19–21), while others a pro-inflammatory effect (22, 23). The influence of various B cell subgroups in MS is supported by clinical trial data, which demonstrates a reduction in relapses in RRMS patients treated with anti-CD20 antibodies (24) and an increased relapse rate after depletion of plasma cells and late stage B cells (23). In PPMS, the success of ocrelizumab in reducing disability progression is likely a result of selective depletion of pro-inflammatory B cell subsets in PPMS patients with MRI evidence of clinically significant ongoing inflammation

## Progressive Ms Pathology and Clinical Characteristics

The pathology of PPMS and SPMS are characterized by widespread diffuse inflammation with slowly expanding lesions, abundant cortical demyelination, brain atrophy, and lymphocyte infiltration and microglial activation in normal appearing white matter (25). In contrast, RRMS is typified by new and active focal inflammatory demyelinating lesions in the CNS white matter. The pathogenic mechanisms underlying PPMS and SPMS are incompletely understood and it remains unclear whether these disease subtypes are caused by similar or unique pathogenic mechanisms (26). Increasing recognition that relapses and MRI-identified lesion activity also occur in some patients with PPMS and SPMS, typically in the early stages of the disease, led to a modification of the phenotypic categories of progressive multiple sclerosis (27). Recent guidelines for diagnosing PPMS and SPMS now include two qualifiers: (1) with or without disease activity, defined by MRI or clinical evidence of inflammatory lesions or relapses; and (2) with or without progression, defined as gradual worsening disability independent of relapses (27). There are multiple areas of clinical and pathological overlap between the different disease subtypes which have led researchers to hypothesize that two distinct yet related pathophysiologic mechanisms are driving the phenotypic differences seen in these subtypes of MS (28, 29). More specifically, one emerging concept is that relapses and remissions, characteristic of RRMS, are caused by an inflammatory process driven by autoreactive effector T cells, while progressive accumulation of disability without remission, characteristic of SPMS and PPMS, is the result of a neurodegenerative process driven by dysfunction of the innate immune system and B cells (30).

There are now over 20 FDA approved disease modifying agents for MS, with one designated by the FDA as an effective option for PPMS. Ocrelizumab is the only FDA approved medication for PPMS, having been approved in early 2017 (31). Ocrelizumab is a monoclonal antibody that targets CD20, a cell marker found principally on B cells (24). The mechanism of action of ocrelizumab is considered to be mainly anti-inflammatory via selective depletion of B cells. In a randomized double-blinded, placebo-controlled trial in patients with PPMS, ocrelizumab reduced confirmed disability as defined by slowed advancement in the expanded disability status scale (EDSS) (31). Prior to this, numerous other immune-targeting therapeutic drugs approved to treat RRMS had been trialed in PPMS without success. Interferon β-1a (32) and β-1b (33), fingolimod (34), rituximab (35) and glatiramer acetate (36) were ineffective at reducing disability accumulation in PPMS. Dronabinol (37) and high dose biotin (38) were also trialed in PPMS and SPMS with the hope that these drugs would promote neuroprotection and repair. Dronabinol showed no significant change in disease worsening, whereas high dose biotin demonstrated disability improvement in 12.6% of patients compared with 0% of the placebo arm in a randomized double-blinded placebo controlled study (38). However, a phase III clinical trial of high dose biotin in the treatment of PPMS and SPMS demonstrated that high dose biotin failed to meet its primary and secondary endpoints, including improvement of disability or progression of disability (39). Research into the mechanisms by which biotin may exert a beneficial effect in progressive MS is ongoing (40). Teriflunomide (41), natalizumab (42), alemtuzumab (43), mitoxantrone (44), and hematopoietic bone marrow transplantation (45, 46) have also been found to alter B cells in MS patients but have not been tested in large scale clinical trials for PPMS. It is not currently known whether PPMS is pathogenetically distinct from RRMS and SPMS but the clinical success of Ocrelizumab in PPMS, viewed in the context of the failure of other disease-modifying therapies, implies a difference in the disease mechanism of PPMS. The mechanism or mechanisms by which B cell depletion produces a therapeutic effect in PPMS will be explored herein.

## Evidence for a Pathogenic Role Of B Cells in PPMS

### Oligoclonal Bands

The presence of unique oligoclonal bands (OCBs) and increased intrathecal IgG synthesis by antigen experienced B cells has long been recognized as a component of MS (47). OCBs are detectable in ~95% of patients upon first presentation who subsequently go on to develop MS. Further, OCBs may have prognostic value in determining the likelihood of progression from CIS to multiple sclerosis and disability accumulation (9–11). Preferential expression of variable gene segments in isolated CNS immunoglobulin from patients with MS indicate that immunoglobulins present in the CNS have undergone affinity maturation likely driven by the presence of a specific antigen (48, 49). MS patients with OCBs have a more aggressive disease course than MS patients without OCBs (50). Moreover, in patients with progressive disease, oligoclonal IgM bands in the CSF are linked to faster progression into SPMS (51, 52) and with active inflammation in PPMS (53). These findings indicate a potential pathogenic role for intrathecal immunoglobulins in MS.

### B Cells in the CSF and Peripheral Blood

In healthy patients, B cells are hardly detectable in the CSF, whereas in MS the mean frequency of B cells among CSF leukocytes is about 5% (12, 13). In contrast to B cells in the periphery, most B cells in the CNS are memory B cells, identified by surface expression of CD27. In patients with RRMS, elevated features of neurodegeneration, as revealed by MRI, correlate with increased numbers of peripheral B cells and a higher proportion of activated B cells (14). Patients with SPMS have greater numbers of specific B cell populations in their peripheral blood, notably DC-SIGN^+^ B cells and CD83^+^ B cells (54), which correlate with disease progression. Another study found that CD19^+^ B lymphocytes expressing TNFα in the periphery are increased in patients with PPMS compared to patients with SPMS, RRMS, or healthy controls (15). CD19^+^ B lymphocytes from RRMS and SPMS patients display hyper-phosphorylation of p65 (55), but this hyperactivity has not been confirmed in PPMS. In addition, anti-inflammatory “regulatory B cells”, which produce IL-13, IL-10, and TGF-β, are reduced in all subtypes of MS compared to healthy controls. Multiple studies have reported flow cytometric characterization of the phenotype of both CSF and peripheral immune cells that offer insight into the possible underlying mechanisms leading to B cell proliferation and activation in MS. Overall, the presence of increased numbers of activated B cells in the CSF and periphery of patients with PPMS and SPMS, the lack of regulatory B cells in all forms of MS, and the correlation of increased B cell populations with disease progression in patients with SPMS indicate a unique role for B cells in the pathology of progressive MS.

### Ectopic Lymphoid Follicles

The CNS is separated physically from the peripheral circulation by the blood brain barrier (BBB), which compartmentalizes the CNS and restricts leukocyte migration into the brain and spinal cord. Historically, the CNS was believed to be an immune-privileged site, but recent evidence has demonstrated a steady trafficking of memory T cells between the periphery and the CNS (56). It is hypothesized that memory T cells enter the CSF using specific adhesion molecules, chemokines, and chemokine receptors and enter the CSF through the epithelium of the choroid plexus (56). These memory T cells then circulate through the CSF and interact with CNS myeloid antigen presenting cells (APCs) within the subarachnoid space surrounding the leptomeninges where they may propagate an immune response. Diverse immune cell infiltrates have been observed in the leptomeninges in patients with RRMS, SPMS, and PPMS (17, 18). Numerous studies have linked these immune cell infiltrates to demyelination and neuronal degeneration in the adjacent cortex (18, 57) leading researchers to postulate that leptomeningeal inflammation is a potential driver of disease progression in MS (16). The spectrum of leptomeningeal inflammation exhibits significant variability, ranging from disorganized collections of immune cells in some patients to well-organized collections of immune cells with many features similar to lymphoid tissue in others (58). These well-organized immune cell structures have been termed ectopic lymphoid follicle-like structures (ELFs) and are characterized by separate B and T cell regions, a network of follicular dendritic cells, plasma cells, and proliferating B cells with evidence of ongoing germinal center reactions (59). While the true incidence and significance of these ELFs in MS patients is still under heavy scrutiny, they are not uncommon in progressive forms of MS. One autopsy study found evidence of ELFs in the meninges in up to 40% of patients with SPMS, but not in RRMS or PPMS (59). Further autopsy series of patients with MS have supported the presence of meningeal ELFs in patients with SPMS (17). Notably, the presence of ELFs is linked with increased cortical demyelination (17).

Further studies looking exclusively at PPMS found no proof of ELFs but instead evidence of more widespread disorganized leptomeningeal inflammation (16). In an autopsy series of 26 patients with PPMS, formal organization of ELFs was not detected; however, a subset of PPMS patients had extensive meningeal immune cell infiltration, consisting of both B and T cells. Patients with evidence of widespread leptomeningeal inflammation had a more severe clinical course and increased cortical demyelination. Further investigation corroborated these findings and demonstrated the presence of ELFs in patients with SPMS and generalized leptomeningeal inflammation in patients with PPMS and RRMS (60). Interestingly, in progressive patients, leptomeningeal inflammation is only present in patients with pathologically active disease defined as the presence of classically active or slowly expanding lesions at the time of autopsy. Patients with pathologically inactive plaques do not display features of leptomeningeal inflammation (60). The makeup of the leptomeningeal immune cell infiltrate varies by disease subtype, with an increased prevalence of plasma cells in patients with either PPMS or SPMS. Additionally, progressive patients with pathologically inactive disease have levels of overall leptomeningeal inflammation similar to those of healthy controls but still have a modest but significantly higher number of plasma cells and overall B cells (60). Leptomeningeal inflammation, given that it is more prevalent in the subset of patients with PPMS who had active disease and can be visualized on MRI (60, 61), may serve as a potential biomarker to identify patients with PPMS who may benefit most from B cell therapy.

Given the correlation between both ELFs in SPMS and widespread disorganized leptomeningeal inflammation in PPMS with adjacent cortical pathology, it is possible that leptomeningeal inflammation is an independent driver of disability, particularly in progressive MS (16, 17). However, the specific role of leptomeningeal inflammation in MS pathogenesis remains an area of active debate. Some studies have described extensive subpial demyelination in patients with PPMS and SPMS without convincing evidence of ELFs or B cell infiltration (62). This seems to indicate that leptomeningeal inflammation with ELFs or B cells may not be needed for cortical demyelination observed in these patients. Additionally, given that most research data on leptomeningeal inflammation in MS comes from autopsy series, the possibility that the leptomeningeal inflammation is a secondary response to primary cortical demyelination rather than a causative factor remains.

## Mechanism of Action of B Cell Mediated Disease Progression in PPMS

### Antibody Production

The presence of unique oligoclonal bands in the CSF of MS patients led to the hypothesis that B cells could be contributing directly to MS pathogenesis via autoantibody mediated CNS tissue damage (47). This idea is supported by the presence of CNS B cell clonal populations in patients with MS that demonstrate evidence of somatic hypermutation and antigen driven affinity maturation (48, 49). Additionally, plasma cells isolated from the CSF of MS patients produce antibodies that make up oligoclonal bands (63). Compared to RRMS patients, SPMS and PPMS patients have higher amounts of plasma cells in perivascular and meningeal immune cell infiltrates indicating a unique role of plasma cells in progressive disease (60). Early studies exploring the role of antibodies in MS pathogenesis demonstrated antibodies bound to disintegrating myelin in acute MS lesions at autopsy and in the marmoset model of EAE (64). Immunoglobulins bound to myelin could induce tissue damage via complement activation (65), activation of microglia/macrophages via activating Fc receptors (66), disturbance of oligodendrocyte physiology (67), or by proteolytic activity on myelin basic protein (68). Additionally, the number of antibody-secreting plasma cells increases with age in patients with PPMS and SPMS (60). Overall, these data indicate that CNS plasma cell antibody production could be playing a role in PPMS disease progression.

It should be emphasized that no specific self-antigen has yet been identified that has consistently been verified as an autoantibody target in MS (69). Evidence supporting intrathecal antibody-mediated injury derives from a study involving adoptive transfer of Ig from the CSF of PPMS patients to naïve mice. These mice succumbed to motor deficits paralleled by CNS pathology, including demyelination and axonal loss within the spinal cord (70). Many potential self-antigens have been implicated by the presence of specific autoantibodies in patients with PPMS. Candidate targets for auto-antibodies in PPMS include anti-neurofilament light (71), anti-ganglioside GM3 (72), and anti-SPAG16 (54). However, these antibodies have not been reliably detected in large populations of PPMS patients, nor has a causal mechanism of injury been well-established. In a study of patients with all subtypes of MS, antibodies specific to KIR4.1 (an ATP-sensitive inward rectifying potassium channel expressed found primarily on glial cells) were found in roughly half of the subjects. However, the presence of anti-KIR4.1 antibodies did not correspond to a specific MS phenotype (73) and subsequent studies have failed to reproduce these findings (74). Overall, while many autoantibodies have been identified in patients with PPMS, no specific autoantibody has been reliably linked to CNS damage.

Clinical data from anti-CD20 treatment of patients with MS argues against a link between treatment benefit and antibody production. B cells down-regulate CD20 expression as they develop into plasma cells and thus mature plasma cells secreting antibodies do not express CD20 (75). Therefore, plasma cells are not directly targeted by ocrelizumab or rituximab and anti-CD20 therapies are unlikely to have a direct impact on intrathecal antibody levels, at least in the short term. This is supported by a lack of measurable change in total serum antibody levels in MS patients treated with rituximab, even in those patients experiencing clinical benefit (76). Additional clinical studies specifically evaluating rituximab's effect on antibody levels have confirmed that rituximab does not change peripheral antibody levels (77). Further, CSF IgG levels, IgG index and oligoclonal band numbers are also unchanged in patients with RRMS treated with rituximab, even in the presence of depleted CSF B and T cells (78). Given that anti-CD20 therapy depletes the vast majority of plasma cell precursor cells, it's possible that long-term CD20-targeted B cell depletion therapy may impact plasma cells in treated patients and thereby alter antibody levels, but antibody modulation does not appear to contribute to the clinical benefit seen shortly after treatment in MS.

### Cytokine Production

B cells exert both pro-inflammatory and anti-inflammatory effects depending on distinct cytokine production (79). B cells are capable of controlling the polarization of effector T cell responses and the formation of memory T cells through cytokine secretion (79). A subset of B cells exhibits anti-inflammatory properties through the secretion of IL-10, TGF-β and IL-35. These unique B cells are identified by CD markers CD19 and CD138 and have been termed “regulatory B cells” due to their hypothesized role in the production of these anti-inflammatory cytokines (79, 80). B cells also produce cytokines that induce T-cell differentiation toward Th1, Th2, or Th17 subtypes (81) and exert an anti-inflammatory role in mouse models of autoimmunity (80).

Patients with RRMS and SPMS have a dysregulated cytokine network, specifically demonstrating a decrease in the anti-inflammatory cytokine IL-10 (82). B cells (particularly memory B cells) isolated from individuals with RRMS and SPMS can also be activated to produce abnormally high amounts of the cytokines TNF-α, LT-α, IL-6, and GM-CSF (82, 83). A study on the peripheral blood of MS patients demonstrated that peripheral pro-inflammatory B cells, defined by the cell surface marker CD19 and by secretion of the cytokine TNF-α, are significantly increased in all subtypes of MS, particularly those with PPMS (15). Additionally, peripheral B regulatory cells, identified by the cell surface marker CD19 and secretion of the cytokines IL-10 and TGF-β, are reduced in all subtypes of MS, particularly those with PPMS. The overproduction or underproduction of specific cytokines by B cells could play a causal role in the pathogenesis of PPMS.

### B Cell Production of LTα

LTα is secreted by B and T cells and binding of membrane bound LTα to follicular dendritic cells induces CXCL13 production (84). CXCL13 is a ligand that binds to the chemokine receptor CXCR5, which is expressed on virtually all B cells, a subset of T cells, and transiently on T cells upon activation (85, 86). CXCL13 is presumed to be a potent chemoattractant that plays a causative role in T and B cell CNS infiltration and lesion formation in MS (87) and is locally produced in active demyelinating MS lesions (87). Elevated CSF CXCL13 also correlates with an increased risk of relapse and unfavorable prognosis in patients with RRMS (88). Elevated levels of CSF CXCL13 increase the likelihood of conversion of CIS to MS (88). In patients with RRMS treated with rituximab, decreased levels of the chemokine CXCL13 correlate with decreased levels of T cells (89). This led study researchers to hypothesize that B cell depletion induces secondary T cell depletion through reduced LT-α-mediated follicular dendritic cell production of CXCL13. Analysis of CSF cytokines has also demonstrated an increase in CXCL13 in patients with PPMS compared to healthy control (90). Additionally, in patients with PPMS, CSF CXCL13 was found to correlate with CSF B and T cell levels (91) and higher amounts of CXCL13 were found in patients with disease activity compared to those without (92). Overall, these data suggest a possible pathogenic role for B cells in PPMS via LT-α and CXCL13, which may be mitigated by anti-CD20 therapies.

### B Cell Production of IL-6

Murine EAE is a commonly used animal model that has been used to decipher the immunopathogenic mechanisms of MS and devise novel therapies (93). EAE is induced by immunizing mice with CNS tissue or myelin peptides in the presence of an adjuvant or by the adoptive transfer of encephalitogenic T cells into naïve mice. Different strains of mice will exhibit different pathology after induction of disease. The SJL/J mouse strain typically demonstrates a relapsing remitting form of demyelinating disease when immunized, whereas C57BL/6 mice display a monophasic or chronic progressive demyelinating disease (94). The latter is considered a suitable model for studying the demyelination and axonal damage present in PPMS and SPMS, although notable differences between murine and human MS disease pathology have raised obvious limitations for the interpretation of EAE results (94).

B cells from mice with EAE produce more IL-6 than naive mice and treatment with monoclonal anti-CD20 antibodies leads to normalized B cell production of IL-6 (95). Genetic deletion of IL-6 exclusively in B-cells during EAE demonstrates a more indolent course compared to control mice without B cell IL-6 deletion (95). In co-culture, B cells enhance Th1 and Th17 T cell responses to fungal infection *in vitro*, partly through IL-6 signaling (96). Additionally, analysis of CSF from patients with PPMS and RRMS revealed that patients with PPMS have significantly higher levels of intrathecal IL-6 production (97). Recent clinical data demonstrates that treatment of PPMS patients with ocrelizumab leads to a reduction in B cell production of IL-6 which correlates with a shift in T cells to a more anti-inflammatory phenotype (98). The concordance of animal and human studies with clinical data in PPMS patients treated with ocrelizumab offers strong evidence for a role of IL-6 in the pathogenesis of PPMS. Taken together, these findings indicate that B cell production of IL-6 could exert inflammatory damage in PPMS by skewing T cells toward a pro-inflammatory phenotype.

### B Cell Production of IL-10

IL-10 is a potent immunoregulatory molecule that is dysregulated in several autoimmune diseases, such as inflammatory bowel disease, rheumatoid arthritis and systemic lupus erythematous (99). Selective genetic deletion of IL-10 in B cells during EAE results in a non-remitting disease course believed to be driven by increased Th1 cell activity (100), supporting an IL-10-mediated anti-inflammatory effect of B cells. Disease is suppressed in EAE mice that received IL-10-producing B cells (101). A distinct subpopulation of B cells, termed B10 cells, potentially function as negative regulators of inflammation and autoimmunity (80). B10 cells have been isolated in the peripheral blood of patients with PPMS, RRMS, and SPMS (102) leading to the hypothesis that deficient functioning of this B cell population may be driving MS pathogenesis (82). What remains unclear is the role of B10 cells in progressive disease; a specific function of B10 cells (or lack thereof) has not been detailed in studies on PPMS to date. Given that the evidence for the anti-inflammatory role of B cell derived IL-10 in PPMS comes primarily from animal studies, it remains to be seen whether these findings will be observed in patients with PPMS and therefore it's specific role in the pathogenesis of PPMS remains unclear.

### Reconstitution of Anti-inflammatory B Cell Population

In addition to the immediate effects of anti-CD20 therapies on patients with PPMS there is also the potential for more long-lasting effects from treatment, specifically through reconstitution of an anti-inflammatory B cell population that may further modulate disease progression and/or activity. Treatment of RRMS patients with rituximab leads to reconstitution of B cells producing lower levels of GM-CSF and higher levels of IL-10 (83). This suggests a durable effect of rituximab on the immunologic underpinnings of MS pathogenic processes. It remains to be seen whether such anti-inflammatory B cell reconstitution occurs in PPMS patients treated with ocrelizumab.

### Antigen Presentation to T Cells

B cells are extremely potent APCs for T cells. They selectively internalize antigen bound to surface immunoglobin and then present this to T cells via MHC II molecules. The antigen concentration necessary for selective internalization and presentation by B cells are 103- to 104-fold lower than those required for presentation by monocytes (103) which potentially makes B cells a necessary APC for T cell activation when antigen levels are low (104). B cells are also more effective APCs when they recognize the same antigen as T cells (103).

The relevance of B cell antigen presentation to MS pathogenesis was initially explored in EAE mouse models. Mice with selective deficiency of MHC II molecules on B cells are resistant to EAE (105). In contrast, mice selectively expressing MHC II only on MOG specific B cells and no other APCs are susceptible to EAE (105). This suggests a causal role of B cells in MS pathogenesis through a mechanism of antigen presentation enhanced by a cognate antigen between B and T cells. In a study exploring the role of B cells in mice with EAE induced by recombinant MOG protein, which produces what is considered a “B cell dependent” EAE mouse model, anti-CD20 treatment reduces Th1 and Th17 subsets significantly more than in the EAE model induced by immunization with MOG peptide residues 35–55 (106). This indicates that B cells, via antigen presentation, may induce a pro-inflammatory polarization with an increase in Th1 and Th17 subsets.

The antigen presentation function of B cells has been explored further in recent human studies. *In vitro* T cell proliferation was found to be increased in RRMS patients with the HLA-DR15^+^ risk haplotype compared to those RRMS patients without the risk haplotype (107). Given that the HLA-DR15 gene encodes a distinct MHC II, this data led to the hypothesis that the increased risk of MS with this haplotype is a direct consequence of antigen presentation by B cells. The study further explored the pathogenicity of the HLA-DR15^+^ haplotype and found that *in vitro* proliferation of T cells was dependent on co-culturing with B cells. When HLA-DR expression by B cells was inhibited by ibrutinib, T cell proliferation was decreased, implying an HLA-DR dependent mechanism of T cell activation by B cells. Additionally, in RRMS patients treated with rituximab, *ex vivo* proliferation and production of pro-inflammatory cytokines by T cells was substantially reduced. The addition of autologous CD20^+^ B cells obtained pre-treatment with rituximab was found to restore CD4^+^ T cell proliferation. Memory B cells, specifically un-switched memory B cells, were the B cell population most strongly correlated with T cell proliferation (107). A recent pathological study demonstrated that PPMS patients had higher amounts of B cells within their CNS lesions compared to patients with RRMS (108). Additionally, lower amounts of B cells within these lesions was correlated with decreased CNS T cell infiltration a better clinical outcome (108). Overall, these data indicate that B cell modulation of T cells via antigen presentation is a likely contributor to MS pathogenesis with memory B cells implicated as the B cell population contributing most to T cell proliferation via antigen presentation. Current research studies have consisted almost exclusively of animal studies and human studies in RRMS and therefore it remains to be seen whether these findings can be replicated in PPMS.

### The Pro-inflammatory and Anti-inflammatory Role of B Cells

The clinical success of ocrelizumab viewed alongside research indicating both pro- and anti-inflammatory effects of distinct B cell populations and cytokines indicates a multi-faceted role of B cells in inflammation. This idea is supported by the pro-inflammatory effects of atacicept in MS, an anti-inflammatory drug previously trialed to treat RRMS (23).

Atacicept is a human recombinant fusion protein that binds to the receptor for both BLyS (B-Lymphocyte Stimulator) and APRIL (A PRoliferation-Inducing Ligand) acting as an antagonist to these ligands and inhibiting receptor activation. These two cytokines are important for B-cell maturation, function, and survival. Atacicept has selective effects on B cells, depleting plasma cells and late stage B cells while sparing B-cell progenitor cells and memory B cells (109). Atacicept is the only immunotherapy for MS whose mechanism of action leads to relative sparing of memory B cells (22).

In a randomized double-blind, placebo-controlled trial of atacicept in patients with RRMS, patients who received atacicept had a higher annualized relapse rate compared to those receiving placebo (23). For this reason, the trial was suspended early and has led to the hypothesis that atacicept's depletion of plasma cells and relative sparing of memory B cells implies that plasma cells mainly function as anti-inflammatory cells while memory B cells are pro-inflammatory in MS (13, 110).

This hypothesis is supported by further data highlighting these distinct functions of plasma cells and memory B cells. In an EAE mouse model, plasma cells from the gut were found to play an anti-inflammatory role on neuroinflammation in EAE through the secretion of IL-10 (19). This fits with previously mentioned data regarding the anti-inflammatory effects of IL-10 in EAE (101) and implicates plasma cells as the B cell subtype responsible for IL-10 secretion. Additionally, immunoglobulin produced by intrathecal plasma cells in progressive multiple sclerosis may have a direct anti-inflammatory effect by binding to inhibitory Fc receptors (111). Oligodendrocyte-specific Igs might also promote remyelination (112). In contrast, memory B cells are likely pro-inflammatory and recent research indicates that *ex vivo* memory B cells play a prominent role in inducing CD4^+^ self-reactivity, likely through a mechanism of antigen presentation (107).

Clinical evidence demonstrating that atacicept increases the rate of MS relapses, taken in conjunction with additional findings suggesting an anti-inflammatory role of plasma cells and a pro-inflammatory role of memory B cells, indicates that B cells can have both a pro and anti-inflammatory effect in MS depending on their specific clonal subset, causing either disease mitigation or progression, respectively.

## Ocrelizumab in PPMS

The success of ocrelizumab at reducing disability in PPMS, in the context of previous failures of other anti-inflammatory drugs approved for RRMS, in particular the anti-CD20 monoclonal antibody rituximab, raises important questions about the specific mechanisms by which ocrelizumab exerts its therapeutic benefit. One hypothesis put forth regarding the success of ocrelizumab and failure of rituximab derives from phenotypic differences in the types of PPMS patients enrolled in each study. Rituximab and ocrelizumab are both CD20 monoclonal antibodies. CD20 is a cell surface marker expressed on most B cell subsets with the exception of early pro-B cells, late stage plasmablasts and terminally differentiated plasma cells (113). In clinical trials, ocrelizumab, but not rituximab, significantly reduced disability progression in PPMS patients (31, 35). In the OLYMPUS trial involving treatment of PPMS patients with rituximab, no significant reduction in disease progression was observed overall (35). However, a subgroup analysis revealed that younger age (<51) and the presence of a gadolinium enhancing lesions on MRI (≥1 gadolinium enhancing lesion at baseline) were predictive of treatment responsiveness (35). In particular, patients who had these characteristics in the placebo arm were 3 times more likely to have clinical disease progression compared to the same demographic of patients treated with rituximab (35). The subsequent ORATORIO trial of ocrelizumab in PPMS was designed with recruitment directed at relatively younger participants (mean age 44.6 years; maximum age 55 years), with shorter disease durations (mean 6.4 years; maximum 15 years), and included a relatively high proportion of participants with gadolinium enhancing lesions at baseline (26%) (31). For comparison, in previous PPMS clinical trials with rituximab, fingolimod, and glatiramer acetate, the percentage of participants with any baseline gadolinium enhancement was 24.5, 13, and 14%, respectively (34–36). In ORATORIO, the subgroup of patients with gadolinium-enhancing lesions at baseline had a greater reduction in risk of disease progression (although the difference was not significant) for those with enhancing lesions (hazard ratio 0.65 [95% CI 0.40–1.06]) vs. for those without enhancing lesions (0.84 [0.62–1.13]) (114). These differences in the patient populations in each study have led to speculation that there are a subset of patients with PPMS, specifically young patients with evidence of active inflammation, who preferentially benefit from B cell depletion therapy due to removal of a B cell-mediated inflammatory effect (115).

A recent retrospective study examined the off-label use of rituximab in the treatment of PPMS and found that 41.5% of PPMS patients treated with rituximab had significant disease progression after 3 years (116). The patients had a higher degree of inflammation prior to treatment as demonstrated by the presence of gadolinium enhancing lesions in 50% of the patients on their baseline brain MRI (116). In contrast, ORATORIO demonstrated a 32.9% incidence of disease progression at 12 weeks with a 26% incidence of gadolinium enhancing lesions on baseline brain MRI (31). The off-label rituximab study had numerous limitations including a retrospective design, which prevented the researchers from including a control group, and a relatively low amount of PPMS patients (43 total) (116). Additionally, it is unclear the specific criteria that led to the off-label use of rituximab and it is likely that the patients were selected for treatment due to rapid disease progression which may have led to a bias selection of patients with a more aggressive form of PPMS. Nevertheless, the study suggests that a significant amount of PPMS patients, despite having evidence of inflammation on their brain MRI, will continue to progress after B-cell depletion with rituximab.

Functional differences in the antibody structure of ocrelizumab compared to rituximab may lead to more favorable safety and tolerability profiles but are unlikely to significantly change the levels of circulating B cells in the periphery and CNS of treated PPMS patients. Rituximab is a chimeric antibody with an Fab domain derived from mouse protein, whereas ocrelizumab is exclusively derived from human protein (117). Compared to rituximab, ocrelizumab has a structurally distinct Fc region domain that binds with higher affinity to natural killer cells. This difference leads to relatively stronger antibody-dependent cell cytotoxicity and relatively weaker complement-dependent cytotoxicity for ocrelizumab compared to rituximab (24). This relative decrease in complement-dependent cytotoxicity is hypothesized to reduce the rate of adverse effects by reducing rates of systemic complement mediated cytokine release (118). Additionally, ocrelizumab has a distinct Fab binding domain that alters its binding affinity to CD20 (119). This difference in epitope binding affinity is unlikely to translate to increased depletion of circulating B cells with ocrelizumab compared to rituximab given that PPMS patients treated with rituximab had near-complete depletion of circulating B cells, defined as a >95% decrease of CD19^+^ B cells, from week 2 to 96 after rituximab treatment (35). Additionally, in RRMS patients treated with rituximab, CSF B cells were reduced by 90% at 24 weeks post-treatment with rituximab (78). Another study examining the efficacy of dual intravenous and intrathecal rituximab for depleting CNS B cells in patients with SPMS found that peripheral B cells were reliably depleted but CSF B cells were incompletely and transiently depleted (120). While it's possible that ocrelizumab may deplete CSF B cells more effectively than rituximab, given that ocrelizumab is administered intravenously it is unlikely to achieve the CNS penetration necessary to outperform intrathecal rituximab administration.

## Conclusion

Ocrelizumab is the first and only FDA approved disease-modifying therapy for patients with PPMS. The characteristics of patients treated in ORATORIO indicate that ocrelizumab likely exerts an anti-inflammatory effect with the most pronounced benefit occurring in younger PPMS patients with a high propensity for disease activity (114). This idea is supported in the rituximab clinical trial in PPMS that showed benefit to a subgroup of younger patients with gadolinium enhancing lesions on MRI (35). Ocrelizumab likely induces an anti-inflammatory effect primarily through abrogating B cell functions, such as cytokine production and antigen presentation. B cells exhibit a spectrum of activity in MS with memory B cells playing a pro-inflammatory role and a subset of B cell lineage cells, such as segments of plasmablasts/plasma cells contributing to the suppression of inflammation. Cytokines produced by B cells, including LT-α, IL-6, and GM-CSF, have been implicated as drivers of the pro-inflammatory effects in MS via T-cell differentiation from naïve T cells into inflammatory Th1/Th17 cells as well as via indirect myeloid cell stimulation of T cells. In contrast, production of IL-10 by B cells may cause an anti-inflammatory effect in PPMS. However, there is currently a lack of clinical human studies to definitively support or refute this claim. B cell antigen presentation also likely plays a prominent role in driving T cell activity by inducing naïve T cell differentiation to Th1/Th17 and driving MS pathogenesis. Ocrelizumab is unlikely to exert benefit in MS through antibody decrement given that immunoglobulin levels remain elevated despite B and T cell depletion in the presence of a treatment benefit. It is unclear if PPMS patients treated with ocrelizumab will experience reconstitution of anti-inflammatory B cells after therapy in a similar way to RRMS patients treated with rituximab (83).

Altogether, the above data indicates that ocrelizumab likely reduces disease progression in PPMS by reducing inflammation. This mechanism of action represents a continuation of the therapeutic paradigm used to treat RRMS in which the primary treatment modality involves drugs that work via reducing inflammation. The benefit of ocrelizumab but the failure of multiple other RRMS anti-inflammatory drugs, in conjunction with the phenotypic differences in PPMS compared to RRMS, has important implications about disease pathogenesis and treatment. Clinical trial data indicates that there is likely a subset of patients with PPMS, typically younger, newly diagnosed patients with gadolinium enhancing lesions on MRI, who have active inflammation contributing to their progressive disability who would benefit from a high potency anti-inflammatory medication. These qualitative differences in subgroups of PPMS patients have implications for the way we classify patients with PPMS. The recent revisions to the classification of MS to include new qualifiers for active disease and presence of progression represents an effort to further delineate PPMS into more clinically useful groups (27). Clinical trials examining the effect of anti-inflammatory treatments on PPMS in patients with or without active disease and with or without progression would shed light on further clinically meaningful phenotypic differences within the PPMS subtype. Leptomeningeal inflammation, given that it is more prevalent in the subset of patients with PPMS who had active disease and that it can be visualized on MRI (60, 61), may serve as a potential biomarker to identify patients with PPMS who may benefit most from B cell therapy. The clinical data also implies that for the majority of patients with PPMS, specifically those older patients without evidence of active disease, further anti-inflammatory treatment is unlikely to influence disease progression. Dedicated research in patients with PPMS without evidence of active inflammation and refinement of MS animal models of neurodegeneration in the absence of inflammation may help elucidate the non-inflammatory, neurodegenerative processes contributing to PPMS disease progression.

Broadening our understanding of disease pathogenesis in PPMS and harnessing that knowledge to develop new and effective treatments represents the next frontier in MS research. This goal carries with it unique challenges given the reduced prevalence of PPMS compared to RRMS and SPMS, making clinical trial recruitment more difficult. Additionally, the EAE mouse model, the most widely studied animal model for MS, is of questionable utility in PPMS given the lack of progressive MS pathologic features (94). Dedicated clinical studies of progressive disease, expanded and novel animal models for progressive disease, and shifting treatment paradigms will hopefully lead to future breakthroughs for patients with PPMS.

## Author Contributions

JH drafted the manuscript. JH, RA, NM, and GW provided the content and edited the manuscript. All authors contributed to the article and approved the submitted version.

## Conflict of Interest

The authors declare that the research was conducted in the absence of any commercial or financial relationships that could be construed as a potential conflict of interest.
